# Effects of green exercise on mental health in older adults: a systematic review and meta-analysis

**DOI:** 10.3389/fpubh.2026.1848669

**Published:** 2026-07-07

**Authors:** Xiaolin Wang, Xuezhen Yang, Yongping Yu, Delong Dong

**Affiliations:** 1 School of Physical Education, Ludong University, Yantai, China; 2 Faculty of Educational Studies, University Putra Malaysia, Serdang, Malaysia; 3 College of Education, Ludong University, Yantai, China

**Keywords:** green exercise, greenspace, mental health, older adults, physical activity

## Abstract

**Introduction:**

This systematic review and meta-analysis aimed to examine the effects of green exercise on mental health in older adults.

**Methods:**

A systematic literature search of PubMed, Web of Science, EBSCOhost, PsycINFO, and the Cochrane Central Register of Controlled Trials identified 26 studies involving 1,468 older adults. Meta-analyses were performed using random-effects models, and risk of bias was assessed using the revised Cochrane Risk of Bias tool. Additionally, subgroup analyses were conducted to explore potential moderators, including geographic region, comparator condition, and intervention characteristics.

**Results:**

The results showed that green exercise significantly improved mental health in older adults compared with indoor exercise [SMD = 0.70, 95% CI (0.44, 0.96)], urban exercise [SMD = 0.79, 95% CI (0.44, 1.15)], and waitlist control conditions [SMD = 0.48, 95% CI (0.14, 0.81)]. Subgroup analyses did not identify significant moderators of the observed effects.

**Conclusion:**

Green exercise may be an effective approach for improving mental health in older adults. These findings highlight the importance of environmental context in shaping the mental health benefits of physical activity in later life and support the potential value of green exercise as a strategy for promoting psychological wellbeing in aging populations.

**Systematic review registration:**

https://www.crd.york.ac.uk/PROSPERO/view/CRD420261277519.

## Introduction

Promoting mental health in older adulthood has become an urgent public health priority as populations age worldwide. By 2030, one in six people will be aged 60 years or over, and by 2050 the global population of older adults is projected to reach 2.1 billion (1). This demographic shift is placing increasing pressure on healthcare systems, not least because depression, anxiety, and psychological distress in later life substantially reduce quality of life and are associated with increased morbidity and mortality (2, 3). Identifying effective and accessible strategies to support mental health in this population is therefore increasingly important.

In recent years, there has been growing recognition that the environments in which people live and age exert powerful influences on health outcomes. Urbanization has increasingly concentrated older populations in cities, where exposure to environmental stressors such as air pollution, noise, overcrowding, and limited access to restorative spaces may increase psychological distress (4, 5). In contrast, a substantial body of evidence suggests that exposure to natural environments confers multiple health benefits, including reduced physiological stress, enhanced mood, and improved cognitive function (6, 7). These findings have stimulated growing interest in nature-based health interventions as potential strategies for promoting healthy aging.

Green exercise, which involves physical activity undertaken in natural or green environments, has attracted increasing attention as a health-promoting behavior that integrates physical activity with exposure to nature (8, 9). Its relevance for older adults lies not only in the independent benefits of exercise and nature exposure, but also in the way natural environments may address age-related vulnerabilities that shape mental health. In later life, declines in attentional control, executive function, and cognitive flexibility may increase susceptibility to mental fatigue and reduce the capacity to recover from cognitively demanding daily activities (10, 11). From the perspective of Attention Restoration Theory, natural environments may help compensate for this vulnerability by providing softly engaging stimuli that require less directed attention and thereby support attentional recovery (12, 13). At the same time, aging is often accompanied by changes in autonomic regulation, stress reactivity, sleep quality, and chronic disease burden, which may heighten vulnerability to psychological distress (11, 14). Stress Reduction Theory provides a complementary explanation by suggesting that natural environments can reduce physiological arousal and promote positive affective responses (13, 15). In this sense, green exercise may be particularly suitable for older adults because it combines physical activity with an environmental context that may reduce cognitive load, support stress recovery, and facilitate mood regulation. These combined cognitive, affective, physiological, and social pathways provide a theoretical basis for examining green exercise as a strategy for promoting mental health in older adults (16–18).

Despite robust evidence supporting green exercise for mental health in general adult populations (8, 16), its applicability to older adults, a group with distinct physiological and psychosocial needs, remains unresolved. Age-related declines in physical function, increased vulnerability to social isolation, and the high burden of chronic disease may all shape how older adults respond to green exercise, warranting dedicated investigation (19). Existing reviews have either subsumed older adults within broader analyses without age-specific examination (8, 20, 21) or drawn largely on qualitative and cross-sectional data (9, 22). To date, no meta-analysis has quantitatively synthesized the effects of green exercise on mental health outcomes exclusively in older adult populations, nor systematically examined potential moderators in this population. As a result, there is limited age-specific evidence to guide how green exercise should be incorporated into strategies for healthy aging.

Therefore, this systematic review and meta-analysis aims to quantitatively synthesize the effects of green exercise on mental health outcomes in older adults. It also examines whether these effects vary according to study, intervention, and participant characteristics. By quantifying pooled effects and exploring these potential moderators, this review strengthens the evidence base for considering green exercise in public health strategies to promote mental health in older adults.

## Methods

### Search strategy and study selection

This systematic review and meta-analysis was registered in PROSPERO (protocol CRD420261277519) and conducted in accordance with the updated PRISMA guidelines. Relevant studies published up to February 1, 2026, were identified through systematic searches of PubMed, Web of Science, EBSCOhost, PsycINFO, and the Cochrane Central Register of Controlled Trials, with no language restrictions. The search strategy was developed using the Boolean operators AND and OR to combine terms related to four key concepts: green or natural environments, exercise or physical activity, mental health outcomes, and older adults. An example of the search strategy used in Web of Science is as follows: [Abstract] (green OR outside OR outdoor OR nature OR forest OR park OR garden OR landscape OR plant OR wood OR flower) AND [Abstract] (exercise OR “physical activity” OR “physical fitness” OR walking OR hiking OR cycling OR jogging OR running OR aerobics) AND [Abstract] (psychology OR emotion OR mood OR affect OR mental OR “wellbeing” OR depression OR anxiety OR stress) AND [Abstract] (elderly OR “older adults”). The search syntax and field tags were adapted for each database, and the complete database-specific search strings are provided in Supplementary File 1. All retrieved records were first imported into a reference-management database, where duplicate records were identified and removed. The remaining records were then independently screened by two reviewers based on titles and abstracts. Studies considered potentially eligible were subsequently assessed in full text according to the predefined inclusion and exclusion criteria. Any disagreements during screening or full-text assessment were resolved by a third reviewer. To further ensure the inclusion of relevant studies, the reference lists of included studies and previous reviews were manually examined, and relevant theses and dissertations were also screened.

### Eligibility criteria

Eligibility was established according to the PICOS framework (Population, Intervention, Comparison, Outcome, Study design). Studies were included if they met the following criteria: (1) participants aged 50 years or older; (2) the intervention involved green exercise, defined as structured physical activity conducted in outdoor natural or semi-natural settings where vegetation or natural elements were a central feature of the exercise environment, such as parks, forests, gardens, greenways, or other vegetated open spaces; (3) comparison groups comprising indoor exercise, urban outdoor exercise with minimal or no greenery, or waitlist controls; (4) primary outcomes assessing mental health, including wellbeing and positive and negative affect; and (5) the study used a randomized controlled trial or crossover randomized controlled trial design. This criterion was applied because random allocation reduces selection bias and uncontrolled confounding, thereby providing more robust estimates of intervention effects.

### Data extraction

Two reviewers independently extracted all relevant data, with disagreements resolved by a third reviewer. The extracted outcomes included overall emotional, affective, and wellbeing-related outcomes, such as mood states, positive and negative affect, psychological wellbeing, and quality of life. For each outcome, means and standard deviations for the intervention and control groups were extracted for effect size calculation. The extracted study and participant characteristics included author, publication year, country or region, study design, sample size, age, and gender distribution. Intervention and comparator details were also recorded, including green exercise type, intervention setting, comparator type, duration, frequency, and session length. For crossover randomized controlled trials, only first-period data, defined as outcome data collected before participants crossed over to the alternative condition, were extracted and synthesized. This approach was used to minimize the potential influence of carry-over effects. Data were systematically organized in a pre-specified spreadsheet to ensure accuracy and consistency, with a detailed overview presented in Table 1.

**Table 1:** Characteristics of the included studies.

Studies	Region	Design	Groups	Female %	Mean Age	GE intervention	Outcome measures
Anandh et al. (33)	India	RCT	GE, *n*= 52; WL, *n*= 52	55	69 ± 2.7	Mindful walking in green fields	PANAS
Barton and Griffin (34)	UK	RCT	GE, *n* = 24; WL, *n* = 15	62.3	53.3 ± 14.8	Green-space walking	POMS
Jia et al. (35)	China	RCT	GE, *n* = 10; UE, *n* = 10	NR	61–79	Forest bathing	POMS
de Brito et al. (36)	USA	RCT	GE, *n* =11; UE, *n* = 12	82.6	50 ± 6.5	Arboretum walking	PANAS
Hvid et al. (37)	Denmark	RCT	GE, *n* = 22; WL, *n* = 23	79.0	51.1 ± 10.6	Forest walking	WHO5
Lacharite'-Lemieux et al. (26)	Canada	RCT	GE, *n* = 12; IE, *n* = 11	100	60.7 ± 4.8	Green-park aerobic and resistance training	EFI
Lee (38)	Korea	RCT	GE, *n* = 30; WL, *n* = 31	NR	76.6 ± 6.6	Urban forest therapy	SRI, K-BDI-II
Levinger et al. (39)	Australia	RCT	GE, *n* = 8; WL, *n* = 8	87.5	85.4 ± 5.3	Supervised park exercise	EQ-5D-5L
Li et al. (40)	China	Crossover-RCT	GE, *n* = 24; UE, *n* = 24	54.2	54.6 ± 2.6	Green-space walking	PANAS
Lim et al. (41)	Korea	RCT	GE, *n* = 30; WL, *n* = 25	NR	74.1	Forest therapy	KGDS
Mao et al. (42)	China	RCT	GE, *n* = 12; UE, *n* = 12	NR	67.2 ± 3.6	Forest walking	POMS
Mao et al. (57)	China	RCT	GE, *n* = 12; UE, *n* = 12	NR	71.8 ± 4.9	Forest walking	POMS
Marselle and Irvine (43)	UK	Crossover-RCT	GE, *n* = 216; UE, *n* = 44	62	≥55	Walking in parks and nature reserves	PANAS, WEMWBS
Müller-Riemenschneider et al. (44)	Singapore	RCT	GE, *n* = 71; WL, *n* = 74	79	51.6 ± 6.4	Green park exercise	SF-12, K-10, WHO5
Sales et al. (56)	Australia	RCT	GE, *n* = 27; WL, *n* = 21	71	72.6 ± 8.1	Park-based physical exercise	SF-12
Park et al. (45)	Korea	RCT	GE, *n* = 36; WL, *n* = 27	85.7	53.8 ± 9.9	Forest healing program	POMS
Pratiwi and Xiang (46)	Indonesia	Crossover-RCT	GE, *n* = 6; UE, *n* = 6	66.7	70.2 ± 4.4	Park walking	POMS
Rantanen et al. (47)	Finland	RCT	GE, *n* = 56; WL, *n* = 53	90	66.1 ± 5.9	Walking in the harbor and parks	WHOQOL
Roe et al. (48)	USA	Crossover-RCT	GE, *n* = 6; UE, *n* = 5	NR	64.8	Green-park walking	MACL, WEMWBS
Sales (49)	Australia	RCT	GE, *n* = 27; WL, *n* = 21	71	71.4 ± 6.7	Park-based mobility and motor-skills training	SF-12
Song et al. (50)	Japan	Crossover-RCT	GE, *n* = 10; UE, *n* = 10	0	58 ± 10.6	Forest walking	POMS
Shin (51)	Korea	Crossover-RCT	GE, *n* = 5; UE, *n* = 5	20	60.3 ± 10.2	Forest walking	POMS
Teas et al. (52)	USA	Crossover-RCT	GE, *n* = 19; IE, *n* = 19	100	58 ± 4	Walking in a grassy area	PANAS
Watkins-Martin et al. (53)	Canada	Crossover-RCT	GE, *n* = 20; UE, *n* = 17	67.6	49.3 ± 11.0	Nature walking	PANAS
White et al. (54)	UK	RCT	IE, *n* = 37; GE, *n* = 37; UE, *n* = 37	100	50.1 ± 3.7	Cycling with countryside video	PANAS, FS-12
Yi et al. (55)	Korea	RCT	GE, *n* = 20; WL, *n* = 17	49	76.3 ± 5	Forest walking and Qigong	EQ-5D, KGDS

GE, green exercise; IE, indoor exercise; UE, urban exercise; WL, waitlist group. EFI, exercise-induced feeling inventory; EQ-5D, Euroqol 5 dimensions; K-BDI-II, Korean beck depression inventory-II; KGDS, Korean form of geriatric depression scale; MACL, mood adjective check list; PANAS, positive affect and negative affect schedule; POMS, profile of mood states; SRI, stress response inventory; WEMWBS, Warwick Edinburgh mental wellbeing scale; WHO5, world health organization five wellbeing index; WHOQOL, world health organization quality of life.

### Risk of bias and certainty of evidence

The risk of bias of the included randomized and crossover randomized controlled trials was assessed using the revised Cochrane Risk of Bias tool for randomized trials [RoB 2; (23)]. Risk of bias was evaluated across five domains: bias arising from the randomization process, bias due to deviations from the intended interventions, bias due to missing outcome data, bias in measurement of the outcome, and bias in selection of the reported result. Each domain and the overall risk of bias were judged as “low risk of bias,” “some concerns,” or “high risk of bias.” The overall certainty of the body of evidence was further assessed using the GRADE framework (24). The assessment considered risk of bias, inconsistency, indirectness, imprecision, and potential publication bias for each main outcome. Two reviewers independently conducted the assessment, and any disagreements were resolved by a third reviewer.

### 2.5 . Data analyses

Meta-analyses were performed using R software (version 4.3.0) within R Studio (version 2023.06.1+524). Separate meta-analyses were conducted for each comparator condition: green exercise vs. waitlist control, green exercise vs. urban exercise, and green exercise vs. indoor exercise. Effect sizes were calculated as standardized mean differences (SMDs; Hedges' g) because the included studies used different instruments with different scoring ranges to assess mental health outcomes. Random-effects models were used to pool study-specific effect sizes within each comparison, accounting for expected between-study variability. Effect sizes were interpreted as trivial (< 0.2), small (0.2–0.5), medium (0.5–0.8), or large (>0.8). Statistical heterogeneity was assessed using the I^2^ statistic, with thresholds of 25%, 50%, and 75% representing low, moderate, and high heterogeneity, respectively. Sensitivity analyses were conducted by sequentially omitting each study to examine the robustness of the pooled estimates.

Additionally, subgroup analyses were performed, where sufficient data were available, to examine whether effect sizes differed across green space type, intervention duration, intervention frequency, participant age, proportion of female participants, geographic region, outcome measure, and publication year. Green space type was categorized according to the primary intervention setting as forest/woodland, urban/community green space, or mixed green space. Forest/woodland included forest bathing, forest walking, or forest therapy settings; urban/community green space included parks, community green spaces, outdoor exercise parks, and arboretums; and mixed green space was used when interventions involved multiple or unclear green space types. Mean participant age was grouped as 50–64 years and ≥65 years. Intervention duration, intervention frequency, proportion of female participants, and publication year were categorized using the median value within the corresponding comparison. Outcome measures were categorized as PANAS, POMS, or other measures. Statistical significance was set at *p* < 0.05.

## Results

### Study characteristics

**Figure 1:**
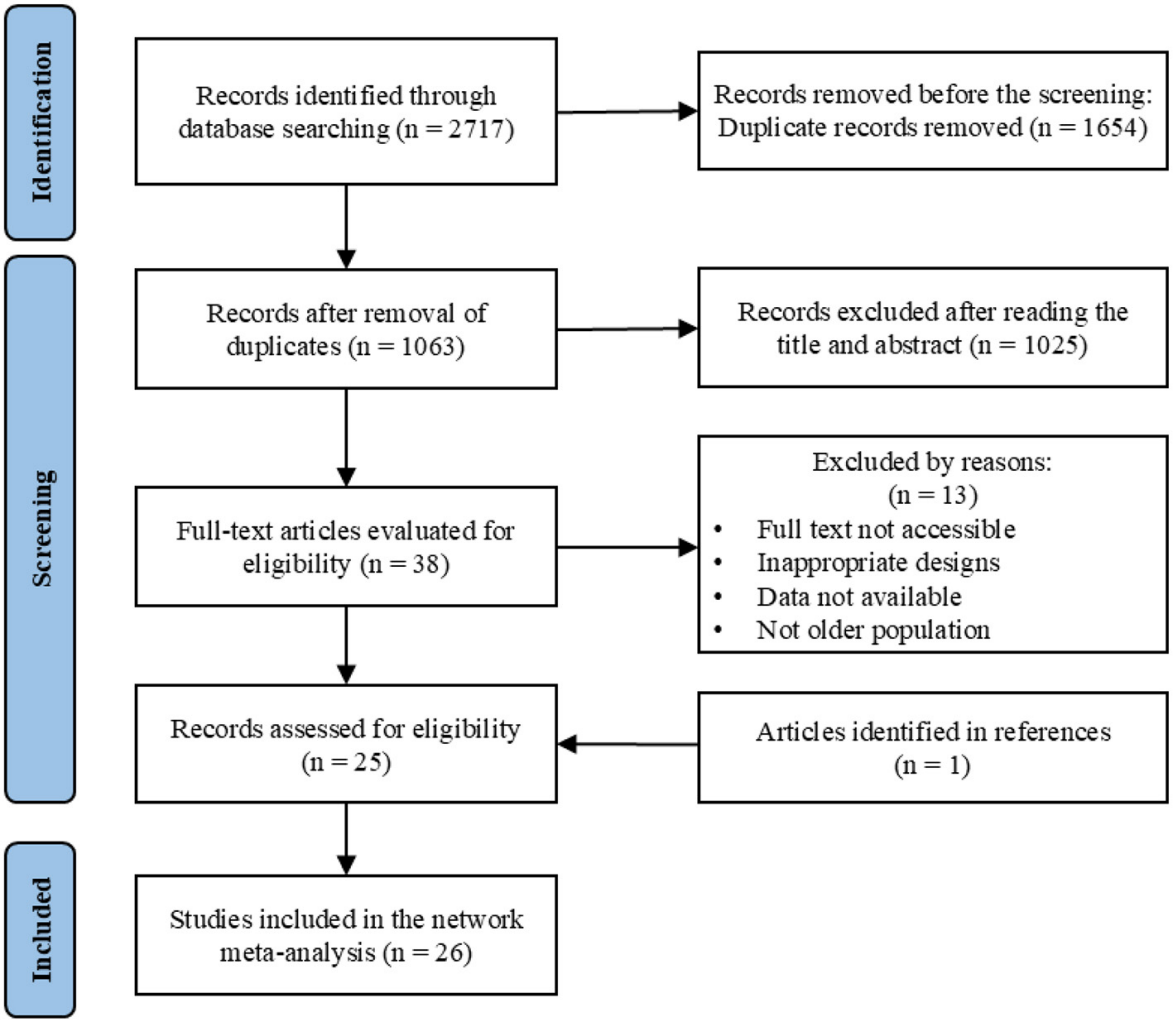
PRISMA flow diagram.

A total of 2,717 records were identified through database searches. After removal of duplicates, 1,063 records were screened, leading to 38 articles being assessed for eligibility. Thirteen studies met the inclusion criteria, with reasons for exclusion of the remaining articles provided in Supplementary File 2. Reference list screening yielded one additional eligible study, resulting in 26 studies included in the meta-analysis. The study selection process is presented in Figure 1.

The main characteristics of the included studies are summarized in Table 1. Across the 26 studies, a total of 1,468 older adults were included, with participant ages ranging from 50 to 86 years. One study focused exclusively on male participants, three on female participants, 18 included both sexes, and four did not report participant sex. Walking was the most frequently applied green exercise intervention (73.1%), followed by combined aerobic–resistance training (15.4%). Mental health outcomes were assessed using a range of validated instruments, most commonly the Profile of Mood States (POMS; 8/26 studies), the Positive and Negative Affect Schedule (PANAS; 7/26), and the 12-Item Short Form Health Survey (SF-12; 4/26).

### Risk of bias and certainty of evidence

The RoB 2 assessment indicated that the overall risk of bias was generally low across the included studies. As shown in the traffic-light plot in Supplementary File 3, most studies were judged to be at low risk of bias, while four studies were rated as having some concerns and two studies were rated as having a high risk of bias. The funnel plot for the overall mental health outcome displayed a generally symmetrical distribution, suggesting a low likelihood of publication bias (Supplementary File 4), which was further supported by Egger's test (*t* = 0.48, *b* = 1.24, *p* = 0.64). According to the GRADE evaluation, the overall certainty of evidence for mental health outcomes was rated as moderate, indicating a moderate level of confidence in the estimated effects of green exercise on mental health outcomes. The certainty rating was mainly influenced by risk-of-bias concerns, particularly unclear reporting of allocation concealment and insufficient information on blinding procedures in several studies. Details of the GRADE assessment are provided in Supplementary File 5.

### Meta-analysis results

**Figure 2:**
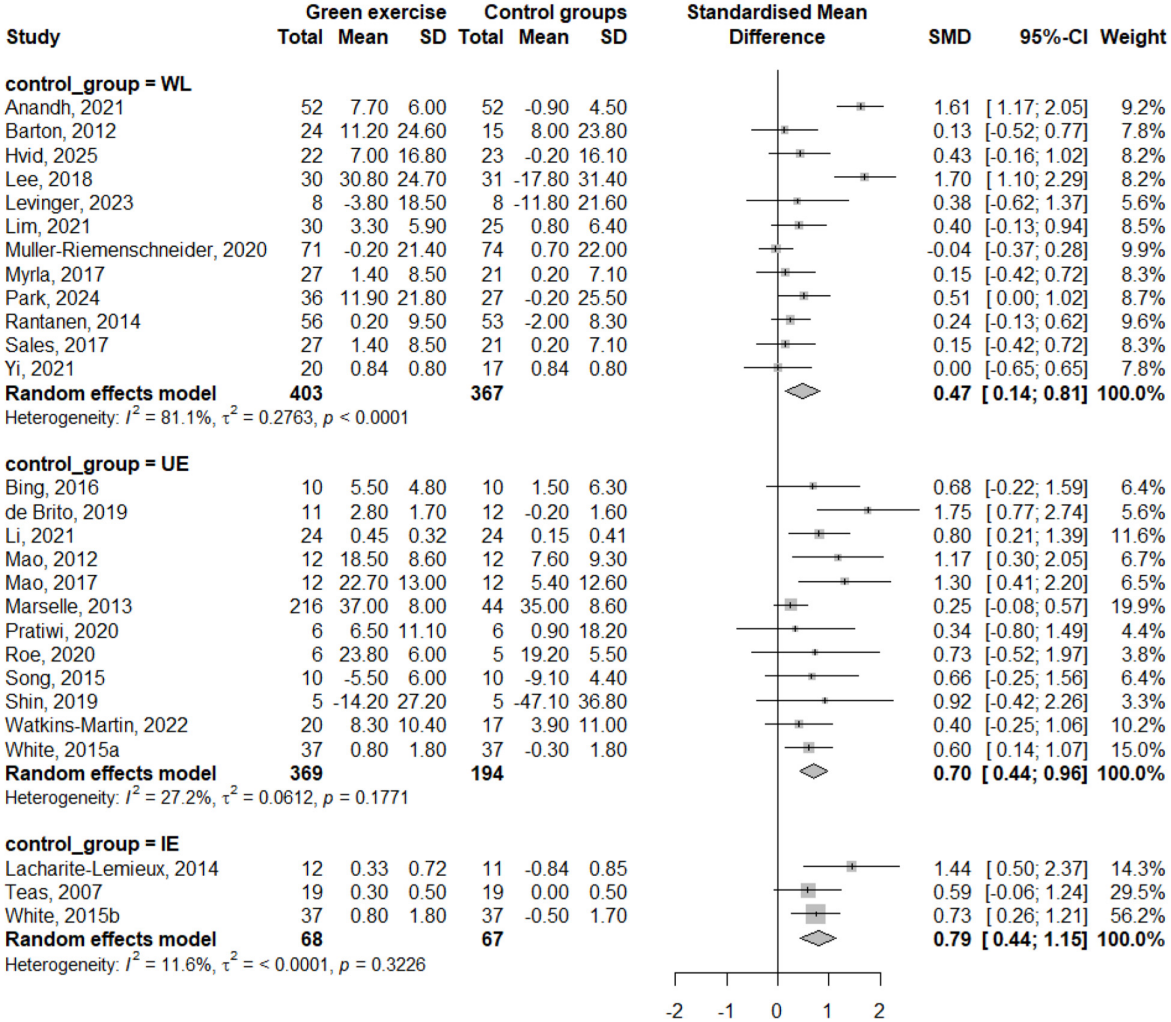
Meta-analysis of the effects of green exercise on mental health. IE, indoor exercise; UE, urban exercise; WL, waitlist group. The bold values represent pooled standardized mean differences (SMDs) and their 95% confidence intervals obtained from the random-effects meta-analysis models for the three control groups (WL, UE, and IE).

The forest plot in Figure 2 presents the pooled effects of green exercise on mental health across three comparison conditions: waitlist control, indoor exercise, and urban exercise. Green exercise was associated with significantly greater improvements in mental health than waitlist control conditions [SMD = 0.48, 95% CI (0.14, 0.81); I^2^ = 81.1%], urban exercise [SMD = 0.70, 95% CI (0.44, 0.96); 27.2%], and indoor exercise [SMD = 0.79, 95% CI (0.44, 1.15); I^2^ = 11.6%]. Sensitivity analyses conducted by sequentially omitting each study showed that no single study substantially influenced the pooled effect, confirming the robustness of the overall estimate. Details are provided in Supplementary File 6. Table 2 presents the results of the subgroup analyses. Only subgroup levels represented by at least three studies were analyzed, ensuring a minimum level of interpretability and stability of the estimates. No significant between-subgroup differences in effect sizes were observed for green space type, intervention duration, intervention frequency, participant age, proportion of female participants, geographic region, or publication year.

**Table 2:** Moderation analysis of green exercise on mental health.

Covariate	*k*	*ES*	95% CI	*I^2^(%)*	*p*-value
Green exercise vs. Waitlist group					
Green space type					0.445
Urban/community	5	0.46	−0.17, 1.09	89.2	
Forest/woodland	5	0.61	0.06, 1.16	77.1	
Mixed	2	0.22	−0.11, 0.54	0	
Training duration					0.672
≤ 8 weeks	5	0.56	−0.02, 1.14	84.0	
>8 weeks	7	0.41	−0.03, 0.84	77.3	
Training frequency					0.671
≤ 1 time/week	7	0.41	−0.03, 0.84	77.3	
>1 time/week	5	0.56	−0.01, 1.13	84.3	
Age					0.191
50–65	4	0.21	−0.09, 0.51	26.8	
>65	8	0.59	0.11, 1.08	84.5	
Female%					0.559
≤ 75%	5	0.43	−0.19, 1.05	86.7	
>75%	5	0.23	−0.02, 0.48	6.8	
Region					0.172
Asia	6	0.69	0.07, 1.31	90.4	
Europe/US	6	0.23	0.01, 0.46	0	
Publish year					0.540
≤ 2020	6	0.37	−0.13, 0.87	81.1	
>2020	6	0.59	0.11, 1.07	78.8	
Green exercise vs. Urban exercise					
Green space type					0.100
Urban/community	5	0.77	0.33, 1.21	28.1	
Forest/woodland	5	0.96	0.53, 1.38	0	
Mixed	2	0.38	0.04, 0.73	34.6	
Training frequency					0.385
≤ 1 time/week	6	0.71	0.42, 0.99	15.5	
>1 time/week	5	0.93	0.51, 1.36	0	
Age					0.230
50–65	7	0.61	0.30, 0.91	40.3	
>65	5	0.94	0.49, 1.38	0	
Female%					0.535
≤ 65%	4	0.51	0.12, 0.90	14.4	
>65%	4	0.71	0.21, 1.22	46.9	
Measures					0.650
PANAS	5	0.62	0.25, 1.00	59.3	
POMS	6	0.88	0.48, 1.28	0	
Others	1	0.73	−0.52, 1.97		
Region					0.353
Asia	7	0.86	0.53, 1.19	0	
Europe/US	5	0.60	0.18, 1.02	54.7	
Publish year					0.633
≤ 2018	6	0.65	0.30, 1.00	38.7	
>2018	6	0.78	0.39, 1.16	11.0	

PANAS, positive affect and negative affect schedule; POMS, profile of mood states.

## Discussion

This systematic review and meta-analysis provides a comprehensive quantitative synthesis of the evidence on the effects of green exercise on the mental health of older adults. The findings demonstrate that green exercise yields significant improvements in mental health compared to indoor exercise, urban outdoor exercise, and waitlist controls. These results are generally consistent with previous literature suggesting that physical activity in natural environments may produce greater psychological benefits than activity undertaken in non-natural settings (8, 9, 25). By focusing specifically on older adults and adopting a comparative framework across different exercise contexts, the present review provides more precise evidence that the mental health benefits of green exercise may derive not only from physical activity itself but also from the restorative properties of natural environments.

The superior mental health outcomes associated with green exercise in older adults can be understood through the synergistic interaction of physical activity and the unique psycho-physiological properties of natural environments. Compared with indoor exercise, natural settings may offer a less monotonous and more affectively supportive context, enhancing affective valence, perceived autonomy, and effortless engagement during physical activity, thereby facilitating emotional recovery (26, 27). The advantage over urban exercise is likely 2-fold. First, it may reduce exposure to common urban stressors, such as traffic noise, air pollution, and crowding, which can themselves contribute to stress responses (28, 29). Second, it adds the restorative properties of natural environments. From a psychophysiological perspective, exposure to natural environments may facilitate more effective stress recovery than urban exposure, as reflected in lower sympathetic activation, greater parasympathetic activity, and reduced cortisol levels (30, 31). This stress-buffering effect may be especially relevant for older adults, who often exhibit age-related declines in autonomic flexibility and physiological recovery capacity (14, 32), making the restorative properties of nature a powerful complement to the general benefits of movement.

The observed effects may be explained by the way green exercise integrates physical activity with restorative environmental exposure. This combination may be especially relevant for older adults, whose mental health is often shaped by cognitive fatigue, reduced physiological recovery capacity, declining mobility, and social isolation (6, 32). Natural environments may make physical activity less monotonous, less cognitively demanding, and more emotionally restorative, while physical activity may make nature exposure more active, embodied, and socially meaningful (9, 11). Thus, the benefits of green exercise are unlikely to reflect a simple additive effect of exercise and nature exposure. Rather, they may arise from a mutually reinforcing process in which restorative environments enhance the experience and sustainability of physical activity, while movement strengthens the psychological value of nature exposure. This interpretation is consistent with Attention Restoration Theory and Stress Reduction Theory, while extending them to the specific psychophysiological needs of older adults (12, 15).

The findings across comparison conditions provide useful context for understanding the potential mechanisms of green exercise. Comparisons with indoor and urban outdoor exercise provide a useful context for interpreting the potential contribution of the exercise environment. Relative to indoor exercise, green exercise may provide a more affectively engaging and restorative context that enhances enjoyment, perceived autonomy, and sustained involvement in activity (26, 27). Relative to urban outdoor exercise, green exercise may involve lower exposure to environmental stressors such as traffic noise, air pollution, crowding, and visually demanding built surroundings, while offering natural features such as vegetation, open space, and natural scenery (28, 29). These environmental characteristics may also support psychophysiological recovery through reduced stress arousal, more favorable autonomic regulation, and lower cortisol responses, which may be particularly relevant for older adults with reduced physiological recovery capacity (30, 31). These findings suggest that the mental health benefits of green exercise may be related not only to physical activity itself or to being outdoors, but also to the natural and stress-reducing features of the activity setting.

Subgroup analyses did not identify significant differences in effect sizes according to green space type, intervention duration, intervention frequency, participant age, proportion of female participants, geographic region, outcome measure, or publication year. These findings suggest that the beneficial effects of green exercise may be relatively consistent across the examined study and intervention characteristics. However, the absence of significant subgroup differences should be interpreted cautiously. Several subgroup comparisons included a limited number of studies, and the included interventions varied considerably in activity type, environmental setting, program structure, session duration, frequency, and outcome measurement. This methodological heterogeneity may have reduced the ability to detect meaningful subgroup patterns and may also help explain why some individual studies reported weaker or non-significant effects despite the overall beneficial findings. Therefore, the current findings should not be interpreted as evidence that intervention characteristics or participant factors are unimportant. Rather, they indicate that existing evidence is not yet sufficient to determine which specific features of green exercise are most strongly associated with mental health benefits in older adults.

These findings have practical implications for promoting mental health in later life. Based on the intervention characteristics and findings of the included studies, green exercise programs for older adults may include walking, light aerobic exercise, or multicomponent exercise delivered in accessible parks, community green spaces, forest or woodland paths, and other safe vegetated environments. When feasible, programs may consider repeated sessions over several weeks, with sessions of approximately 30–60 min conducted once or twice per week, while being adapted to participants' functional capacity, health status, and mobility limitations. For policymakers and urban planners, the findings support the development of age-friendly green spaces, green prescriptions, and nature-based referral pathways that link healthcare services with community-based exercise opportunities. Given the variability in intervention protocols and green space characteristics, these recommendations should be interpreted as practice-oriented guidance rather than definitive prescriptions.

### Limitations

Several limitations should be acknowledged. First, the available evidence was limited in scale, with many individual studies having relatively small sample sizes. This may have reduced statistical power, particularly for detecting smaller effects and subgroup differences, and may limit the generalizability of the findings to broader older adult populations. Second, residual heterogeneity remained in certain comparisons, which may be related to differences in intervention characteristics, implementation settings, and incomplete reporting of intervention protocols. Third, most included studies did not report objective indicators of green exposure, such as vegetation coverage, NDVI, or the degree of naturalness of the intervention setting. Therefore, green exercise was classified according to the reported intervention setting and environmental characteristics, which may have introduced some variability in intervention classification. Fourth, mental health outcomes were assessed using a range of different instruments and covered different aspects of psychological functioning, which may have introduced additional measurement heterogeneity and limited comparability across studies. Fifth, this review was restricted to randomized controlled trials and crossover randomized controlled trials. While this criterion strengthened the internal validity and methodological consistency of the synthesis, some relevant quasi-experimental evidence from applied or community-based settings may not have been included. Future studies should use larger randomized trials with longer follow-up periods, clearer reporting of intervention protocols, objective indicators of green exposure, and standardized mental health outcomes. Further research should also examine whether the effects of green exercise differ across older adults with different health conditions, including those with chronic diseases or poorer baseline mental health.

## Conclusion

This systematic review and meta-analysis indicates that green exercise improves mental health in older adults and provides greater psychological benefits than indoor exercise, urban exercise, and waitlist control conditions. These findings highlight the importance of environmental context in shaping the mental health benefits of physical activity in later life and support the potential value of green exercise as a strategy for promoting psychological wellbeing in aging populations.

## Data Availability

The original contributions presented in the study are included in the article/Supplementary material, further inquiries can be directed to the corresponding authors.
