# Supplementary material for: Effects of green exercise on mental health in older adults: a systematic review and meta-analysis
Source: Front Public Health. 2026 Jul 7;14:1848669. doi: 10.3389/fpubh.2026.1848669 (PMC13384818; doi:10.3389/fpubh.2026.1848669)
Supplement: Supplementary file 1 [file Table_1.docx]

**Supplementary File 1.** Search Alert (1.2.2026)

Pubmed (488)

((green[Title/Abstract] OR outside[Title/Abstract] OR outdoor[Title/Abstract] OR nature[Title/Abstract] OR forest[Title/Abstract] OR park[Title/Abstract] OR garden[Title/Abstract] OR landscape[Title/Abstract] OR plant[Title/Abstract] OR wood[Title/Abstract] OR flower[Title/Abstract]) AND (exercise[Title] OR physical activity[Title] OR physical fit[Title] OR walking[Title] OR hiking[Title] OR cycling[Title] OR jogging[Title] OR running[Title] OR aerobics[Title])) AND (Psychology[Title/Abstract] OR emotion[Title/Abstract] OR mood[Title/Abstract] OR affect[Title/Abstract] OR mental[Title/Abstract] OR well-being[Title/Abstract] OR depression[Title/Abstract] OR anxiety[Title/Abstract] OR stress[Title/Abstract]) AND (elderly[Title/Abstract] OR older adults[Title/Abstract])

Web of Science (909)

[Abstract] (green OR outside OR outdoor OR nature OR forest OR park OR garden OR landscape OR plant OR wood OR flower) AND [Abstract] (exercise OR physical activity OR physical fit OR walking OR hiking OR cycling OR jogging OR running OR aerobics) AND [Abstract] (Psychology OR emotion OR mood OR affect OR mental OR well-being OR depression OR anxiety OR stress) AND [Abstract] (elderly OR older adults)

EBSCOhost (1080)

AB (green OR outside OR outdoor OR nature OR forest OR park OR garden OR landscape OR plant OR wood OR flower) AND AB (exercise OR physical activity OR physical fit OR walking OR hiking OR cycling OR jogging OR running OR aerobics) AND AB (Psychology OR emotion OR mood OR affect OR mental OR well-being OR depression OR anxiety OR stress) AND AB (elderly OR older adults)

PsycINFO (218)

Abstract: green OR Abstract: outside OR Abstract: outdoor OR Abstract: nature OR Abstract: forest OR Abstract: park OR Abstract: garden OR Abstract: landscape OR Abstract: plant OR Abstract: wood OR Abstract: flower AND Abstract: exercise OR Abstract: physical activity OR Abstract: physical fit OR Abstract: walking OR Abstract: hiking OR Abstract: cycling OR Abstract: jogging OR Abstract: running OR Abstract: aerobics AND Abstract: Psychology OR Abstract: emotion OR Abstract: mood OR Abstract: affect OR Abstract: mental OR Abstract: well-being OR Abstract: depression OR Abstract: anxiety OR Abstract: stress AND Abstract: elderly OR Abstract: older adults

Cochrane Central Register of Controlled Trials (22)

[Title Abstract Keyword] (green OR outside OR outdoor OR nature OR forest OR park OR garden OR landscape OR plant OR wood OR flower) AND [Title Abstract Keyword (exercise OR physical activity OR physical fit OR walking OR hiking OR cycling OR jogging OR running OR aerobics) AND [Title Abstract Keyword] (Psychology OR emotion OR mood OR affect OR mental OR well-being OR depression OR anxiety OR stress) AND [Title Abstract Keyword] (elderly OR older adults)

**Supplementary File 2.** References for Excluded Studies

| **Article** | **Reason for exclusion** |
| --- | --- |
| Anzman-Frasca, S., Drozdowsky, J., Zayatz, C. et al. (2023). Effects of a randomized controlled hiking intervention on daily activities, sleep, and stress among adults during the COVID-19 pandemic. BMC Public Health, 23(1), 892. | Not older population. |
| Astell-Burt, T., Feng, X., & Kolt, G. S. (2013). Mental health benefits of neighbourhood green space are stronger among physically active adults in middle-to-older age: evidence from 260,061 Australians. Preventive medicine, 57(5), 601-606. | Inappropriate design. |
| Ayaz, E. Y., Dincer, B., Mete, E. et al. (2024). Evaluating the impact of aerobic and resistance green exercises on the fitness, aerobic and intrinsic capacity of older individuals. Archives of gerontology and geriatrics, 118, 105281. | Data not available. |
| Tu, P. C., Cheng, W. C., Hou, P. C., & Chang, Y. S. (2020). Effects of types of horticultural activity on the physical and mental state of elderly individuals. International Journal of Environmental Research and Public Health, 17(14), 5225. | Non-green exercise. |
| Dickmeyer, A., Smith, J. J., Halpin, S.et al. (2025). Walk‐and‐Talk Therapy Versus Conventional Indoor Therapy for Men With Low Mood: A Randomised Pilot Study. Clinical Psychology & Psychotherapy, 32(1), e70035. | Not older population. |
| Du, M., Wang, Y., Zhang, Y.et al. (2024). How outdoor horticultural activities affect elderly adults’ thermal, physiological and psychological responses: a field study. International Journal of Biometeorology, 68(7), 1-14. | Data not available. |
| Lee, J. Y., & Lee, D. C. (2014). Cardiac and pulmonary benefits of forest walking versus city walking in elderly women: A randomised, controlled, open-label trial. European Journal of Integrative Medicine, 6(1), 5-11. | Data not available. |
| Ojala, A., Korpela, K., Tyrväinen, L.et al. (2019). Restorative effects of urban green environments and the role of urban-nature orientedness and noise sensitivity: A field experiment. Health & place, 55, 59-70. | Not older population. |
| Sung, J., Woo, J. M., Kim, W. et al. (2012). The effect of cognitive behavior therapy-based “forest therapy” program on blood pressure, salivary cortisol level, and quality of life in elderly hypertensive patients. Clinical and Experimental Hypertension, 34(1), 1-7. | Data not available. |
| Tyrväinen, L., Ojala, A., Korpela, K. et al. (2014). The influence of urban green environments on stress relief measures: A field experiment. Journal of environmental psychology, 38, 1-9. | Not older population. |
| Keenan, R., Lumber, R., Richardson, M. et al. (2021). Three good things in nature: a nature-based positive psychological intervention to improve mood and well-being for depression and anxiety. Journal of Public Mental Health, 20(4), 243-250. | Not older population. |
| Wu, Q., Ye, B., Lv, X. et al. (2020). Adjunctive therapeutic effects of cinnamomum camphora forest environment on elderly patients with hypertension. International Journal of Gerontology, 14(4), 327-331. | Full-text not available. |
| Yi, J., Ku, B., Kim, S. G. et al. (2019). Traditional Korean medicine-based forest therapy programs providing electrophysiological benefits for elderly individuals. International Journal of Environmental Research and Public Health, 16(22), 4325. | Data not available. |

**Supplementary File 3.** Revised Cochrane risk-of-bias tool for randomized trials (RoB2) Traffic-light plot


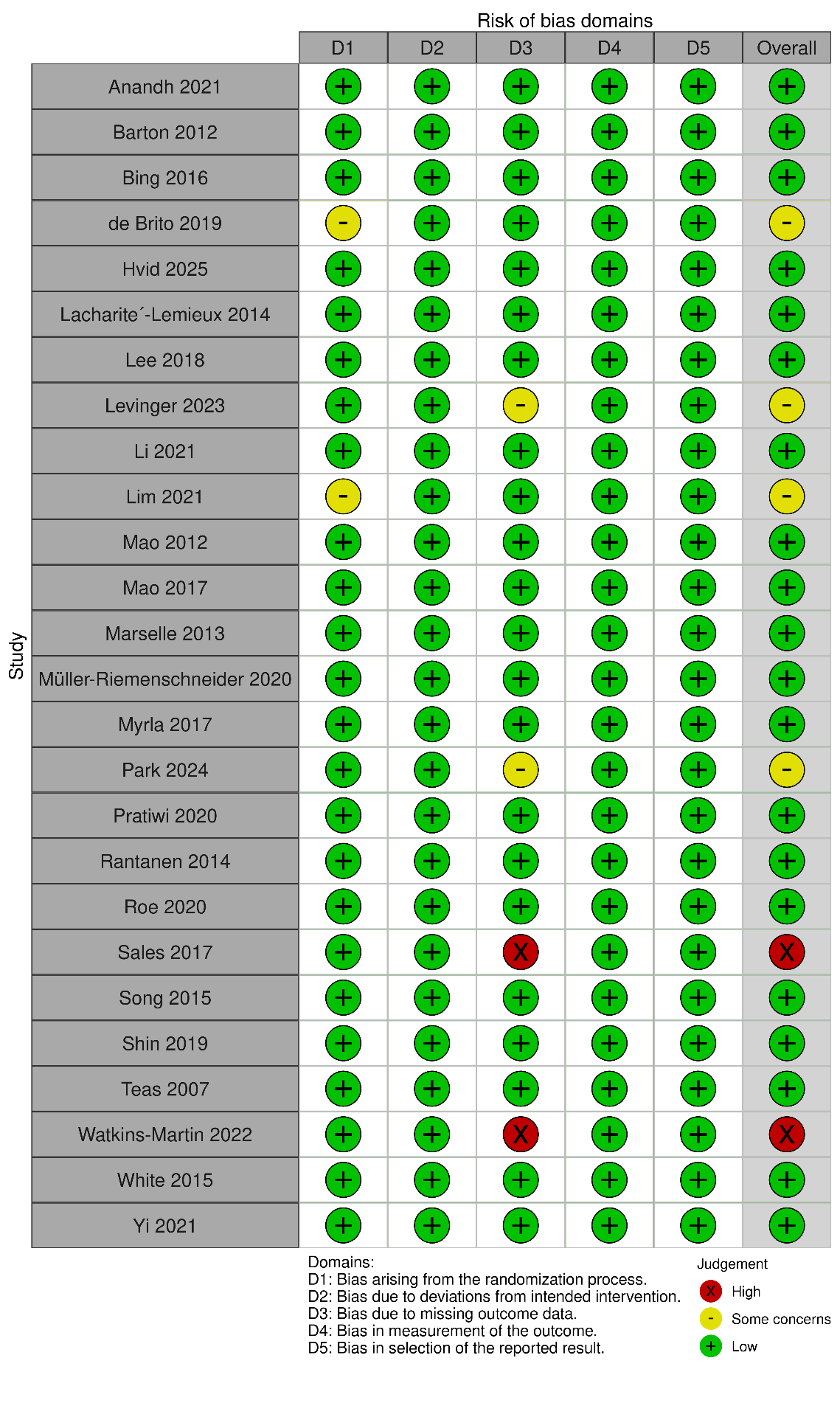


**Figure S1** RoB2 Traffic-light plot

**Supplementary File 4.** Funnel Plots


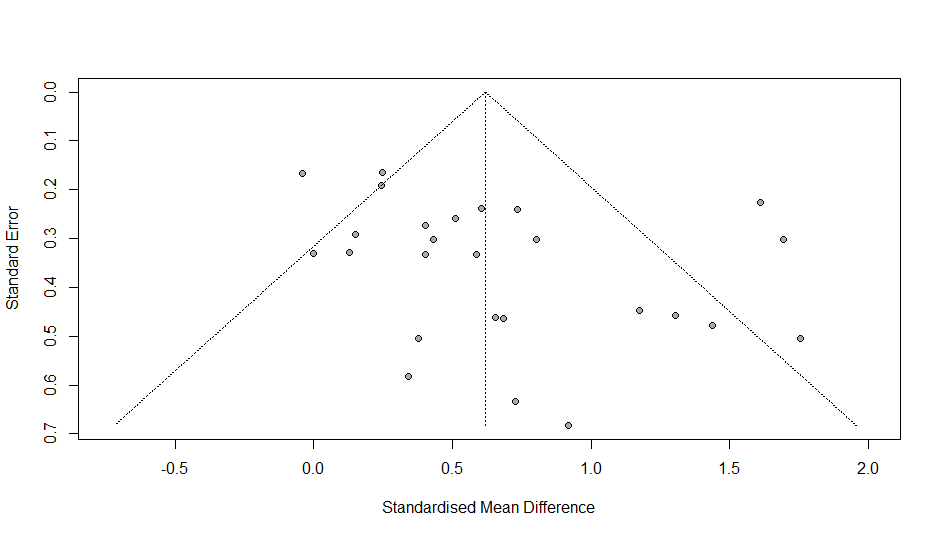


**Figure S1**. Funnel Plots

**Supplementary File 5.**

**Author(s):** Xiaolin Wang, Xuezhen Yang, Delong Dong
**Date:** 2026-01-03
**Question:** Should green exercise be used for mental health?
**Settings:** exercise
**Bibliography:** Anandh et al., 2021; Barton et al., 2012; de Brito et al., 2019; Hvid et al., 2025; Jia et al., 2016; Lacharité-Lemieux et al., 2015; Lee & Son, 2018; Levinger et al., 2023; Li et al., 2021; Lim et al., 2021; Mao et al., 2012; Mao et al., 2017; Marselle et al., 2013; Müller-Riemenschneider et al., 2020; Pratiwi et al., 2020; Rantanen et al., 2015; Roe et al., 2020; Sales et al., 2017; Sales et al., 2015; Shin & Choi, 2019; Song et al., 2015; Teas et al., 2007; Watkins-Martin et al., 2022; White et al., 2015; Yi et al., 2021

| **Quality assessment** | | | | | | | **No of patients** | | **Effect** | | **Quality** | **Importance** |  |
| --- | --- | --- | --- | --- | --- | --- | --- | --- | --- | --- | --- | --- | --- |
|  |  |  |  |  |  |  |  |  |  |  |  |  |  |
| **No of studies** | **Design** | **Risk of bias** | **Inconsistency** | **Indirectness** | **Imprecision** | **Other considerations** | **Green exercise** | **Control** | **Relative (95% CI)** | **Absolute** |  |  |  |
| **Mental Health (Green Exercise vs. Waitlist Group) (Better indicated by higher values)** | | | | | | | | | | | | |  |
| 12 | randomised trials | serious^1^ | no serious inconsistency | no serious indirectness | no serious imprecision | none | 403 | 367 | - | SMD 0.47 higher (0.14 to 0.81 higher) | ⊕⊕⊕O MODERATE | CRITICAL |  |
| **Mental Health (Green Exercise vs. Urban Exercise) (Better indicated by higher values)** | | | | | | | | | | | | |  |
| 12 | randomised trials | serious^2^ | no serious inconsistency | no serious indirectness | no serious imprecision | none | 369 | 194 | - | SMD 0.70 higher (0.44 to 0.96 higher) | ⊕⊕⊕O MODERATE | CRITICAL |  |
| **Mental Health (Green Exercise vs. Indoor Exercise) (Better indicated by higher values)** | | | | | | | | | | | | |  |
| 3 | randomised trials | serious^3^ | no serious inconsistency | no serious indirectness | no serious imprecision | none | 68 | 67 | - | SMD 0.79 higher (0.44 to 1.15 higher) | ⊕⊕⊕O MODERATE | CRITICAL |  |

^1^ 6 studies did not report whether allocation was concealed or whether outcome assessors were blinded.
^2^ 12 studies did not mention whether group allocation was concealed, and most studies did not report blinding information
^3^ 2 studies did not mention whether group allocation was concealed, and most studies did not report blinding information

GRADE Working Group grades of evidence

High quality: Further research is very unlikely to change our confidence in the estimate of effect.

Moderate quality: Further research is likely to have an important impact on our confidence in the estimate of effect and may change the estimate.

Low quality: Further research is very likely to have an important impact on our confidence in the estimate of effect and is likely to change the estimate.

Very low quality: We are very uncertain about the estimate.

**Supplementary File 6.** Sensitivity analysis


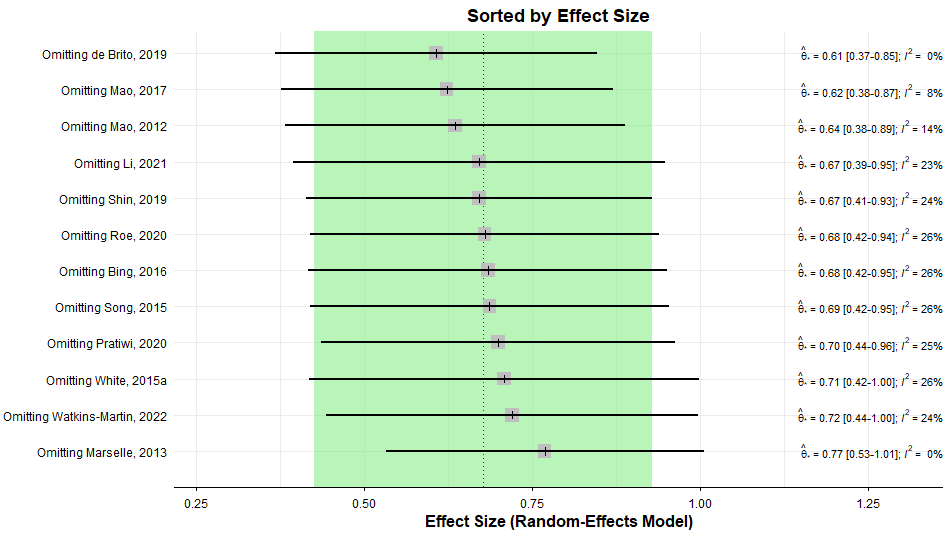

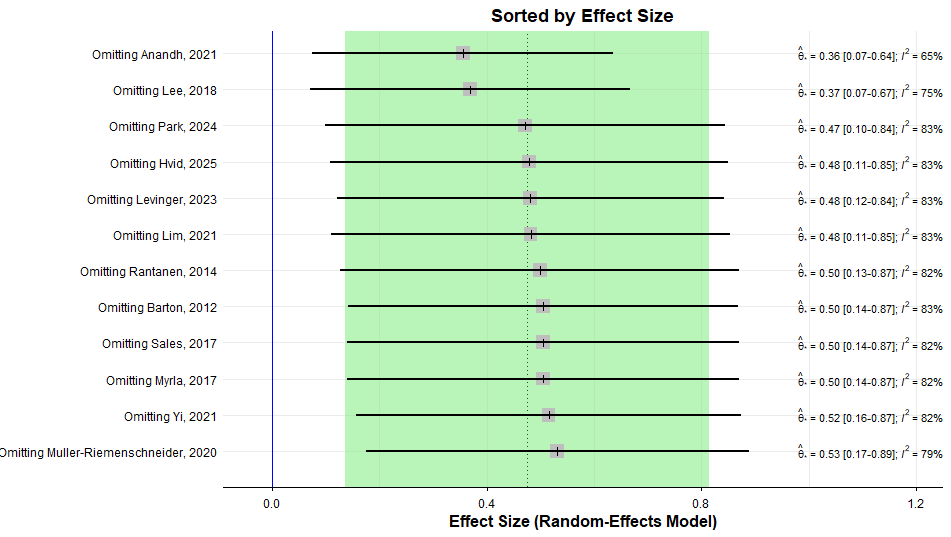


**Figure S2**. Sensitivity analysis results

**Supplementary File 7.** Detailed characteristics of the included studies

| **Studies** | **Design** | **Season** | **Green space type** | **Groups** | **Female %** | **Mean Age** | **Duration** | **Frequency** | **Green exercise component** |
| --- | --- | --- | --- | --- | --- | --- | --- | --- | --- |
| Anandh 2021;  India | RCT | NR | Urban/community green space | GE 52  WL 52 | 55% | 69±2.7 | 8 weeks | 5 times/wk | 20min mindful walking in trees and fields vs. routine control |
| Barton 2012;  UK | RCT | Summer | Mixed green space | GE 24  WL 15 | 62.3% | 53.3±14.8 | 6 weeks | 1 time/wk | Walking in green space |
| Jia 2016;  China | RCT | Summer | Forest/woodland | GE 10  UE 10 | NA | 61-79 | 4 days | 4 times/wk | Forest bathing trip vs. city area |
| de Brito 2019;  USA | RCT | NR | Urban/community green space | GE11  UE12 | 82.6% | 50±6.5 | 3 weeks | 1 time/wk | 50-min walking in an arboretum vs. in a residential development area |
| Hvid 2025;  Denmark | RCT | NR | Forest/woodland | GE 22  WL 23 | 79.0% | 51.1±10.6 | 7 weeks | 2 times/wk | 2-40min forest walking vs. waitlist control group |
| Lacharite´-Lemieux 2014; Canada | RCT | Summer | Mixed green space | GE 12  IE 11 | 100% | 60.7±4.8 | 12 weeks | 3 times/wk | Aerobic and resistance training in green park vs. indoor room |
| Lee 2018;  Korea | RCT | Spring and summer | Forest/woodland | GE 30  WL 31 | NA | 76.6±6.6 | 9 weeks | 1 time/wk | Urban forest therapy program |
| Levinger 2023;  Australia | RCT |  | Urban/community green space | GE 8  WL 8 | 87.5% | 85.4±5.3 | 12 weeks | 1 time/wk | 1.5h structured supervised exercise  program at park |
| Li 2021;  China | Crossover-RCT | Summer | Urban/community green space | GE 24  UE 24 | 54.2% | 54.6±2.6 | 2 days | 1 time/wk | 1.6 km walking in a green space vs. in an urban area |
| Lim 2021;  Korea | RCT | Summer | Forest/woodland | GE 30  WL 25 | NA | ≥ 65 (74.1) | 11 weeks | 1 time/wk | Forest therapy program |
| Mao 2012;  China | RCT | Summer | Forest/woodland | GE 12  UE 12 | NA | 67.2±3.6 | 4 days | 7 times/wk | 3h forest walking vs. city walking |
| Mao 2017;  China | RCT | Summer | Forest/woodland | GE 12  UE 12 | NA | 71.8±4.9 | 4 days | 7 times/wk | 3h forest walking vs. city walking |
| Marselle 2013;  UK | Crossover-RCT | Summer | Mixed green space | GE 216  UE 44 | 62% | ≥ 55 | 13 weeks | NR | Walking in the country park, nature reserve vs. streets, shopping centers |
| Müller-Riemenschneider 2020; Singapore | RCT | Spring and summer | Urban/community green space | GE 71  WL 74 | 79% | 51.6±6.4 | 24 weeks | 1 time/wk | 1h green park exercise (walking, cycling, aerobic, and strength activity) |
| Myrla 2017;  Australia | RCT | NR | Urban/community green space | GE 27  WL 21 | 71% | 72.6±8.1 | 18 weeks | 1 time/wk | Strength, stretching, and flexibility in the park |
| Park 2024;  Korea | RCT | Spring and summer | Forest/woodland | GE 36  WL 27 | 85.7% | 53.8±9.9 | 4 weeks | 2 times/wk | Forest healing program vs. no intervention |
| Pratiwi 2020;  Indonesia | Crossover-RCT | tropical | Urban/community green space | GE 6  UE 6 | 66.7% | 70.2±4.4 | 3 days | 1 time/wk | Walking in the park vs. the city area |
| Rantanen 2014;  Finland | RCT | NR | Mixed green space | GE 56  WL 53 | 90% | 66.1±5.9 | 12 weeks | 1 time/wk | Walking in the harbor and parks |
| Roe 2020;  US | Crossover-RCT | NR | Urban/community green space | GE 6  UE 5 | NA | 64.8 | 2 days | 2 times/wk | Walking in green parks |
| Sales 2017;  Australia | RCT | NR | Urban/community green space | GE 27  WL 21 | 71% | 71.4±6.7 | 18 weeks | 0.5 time/wk | 1-1.5h upper body mobility and fine motor skills training in the park |
| Song 2015;  Japan | Crossover-RCT | Spring and summer | Forest/woodland | GE 10  UE 10 | 0 | 58±10.6 | 2 days | 2 times/wk | 17min walking in the forest vs. city area |
| Shin 2019;  Korea | Crossover-RCT | Spring and summer | Forest/woodland | GE 5  UE 5 | 20% | 60.3±10.2 | 2 days | 1 time/wk | 15min single session walking in the forest and urban environment |
| Teas 2007;  USA | Crossover-RCT | Spring | Mixed green space | GE 19  IE 19 | 100% | 58±4 | 1 week | 1 time/wk | 1h walking in a grassy area vs. in a campus gym |
| Watkins-Martin 2022;  Canada | Crossover-RCT | Spring and summer | Urban/community green space | GE 20  UE 17 | 67.6% | 49.3±11.0 | 2 days | 1 time/wk | 60min nature walking vs urban walking |
| White 2015;  UK | RCT | Winter | Mixed green space | IE 37  GE 37  UE 37 | 100% | 50.1±3.7 | 2 days | 1 time/wk | 15min cycling with countryside video vs. blank wall |
| Yi 2021;  Korea | RCT | Spring | Forest/woodland | GE 20  WL 17 | 49% | 76.3±5 | 6 weeks | 2 times/wk | Walking, Qigong in the forest |

Note: GE, green exercise; IE, indoor exercise; UE, urban exercise; WL, waitlist group. EFI, exercise-induced feeling inventory; EQ-5D, Euroqol 5 dimensions; K-BDI-Ⅱ, Korean beck depression inventory-Ⅱ; KGDS, Korean form of geriatric depression scale; MACL, mood adjective check list; PANAS, positive affect and negative affect schedule; POMS, profile of mood states; SRI, stress response inventory; WEMWBS, Warwick Edinburgh mental well-being scale; WHO5, world health organization five well-being index; WHOQOL, world health organization quality of life.
